# Early-life sleep disruption impairs subtle social behaviours in prairie voles: a pose-estimation study

**DOI:** 10.1098/rsos.230700

**Published:** 2023-07-12

**Authors:** Lezio S. Bueno-Junior, Carolyn E. Jones-Tinsley, Noah E. P. Milman, Peyton T. Wickham, Brendon O. Watson, Miranda M. Lim

**Affiliations:** ^1^ Department of Psychiatry, University of Michigan Medical School, Ann Arbor, MI, USA; ^2^ Veterans Affairs VISN20 Northwest MIRECC, VA Portland Health Care System, Portland, OR, USA; ^3^ Oregon Alzheimer's Disease Research Center, Department of Neurology, Oregon Health and Science University, Portland, OR, USA

**Keywords:** early-life sleep disruption, prairie voles, pair bond formation, cohabitation, pose estimation

## Abstract

Early-life sleep disruption (ELSD) has been shown to have long-lasting effects on social behaviour in adult prairie voles (*Microtus ochrogaster*), including impaired expression of pair bonding during partner preference testing. However, due to the limitations of manual behaviour tracking, the effects of ELSD across the time course of pair bonding have not yet been described, hindering our ability to trace mechanisms. Here, we used pose estimation to track prairie voles during opposite-sex cohabitation, the process leading to pair bonding. Male–female pairs were allowed to interact through a mesh divider in the home cage for 72 h, providing variables of body direction, distance-to-divider and locomotion speed. We found that control males displayed periodic patterns of body orientation towards females during cohabitation. In contrast, ELSD males showed reduced duration and ultradian periodicity of these body orientation behaviours towards females. Furthermore, in both sexes, ELSD altered spatial and temporal patterns of locomotion across the light/dark cycles of the 72 h recordings. This study allows a comprehensive behavioural assessment of the effects of ELSD on later life sociality and highlights subtle prairie vole behaviours. Our findings may shed light on neurodevelopmental disorders featuring sleep disruption and social deficits, such as autism spectrum disorders.

## 1. Introduction

Early-life sleep disruption features prominently in human conditions such as autism spectrum disorders (ASD), a hallmark of which is alterations in social behaviour throughout life [1,2]. Individuals with ASD may exhibit very subtle differences in their social interest during dyadic interactions, including differences in verbal entrainment, non-verbal reciprocity and visual attention [3–5]. We and others have hypothesized that early-life sleep disruption may affect the development of neural pathways related to sociality and exacerbate social deficits in ASD [6]. Highly controlled experimental manipulation using animal models is an important avenue towards improved understanding of the relationship between early-life sleep and later changes in social behaviours in ASD [7]. However, these subtle social differences may be challenging to quantify in conventional laboratory rodents such as mice and rats, whose social interactions are less comparable to those of humans [8].

A wild rodent species, the prairie vole (*Microtus ochrogaster*), offers much better insight into human-like social behaviours. Unlike rats and mice, prairie voles are monogamous, i.e. they show strong affiliations with opposite-sex mates after a period of cohabitation, including in the laboratory [9–11]. After cohabitation, mates display affiliative behaviour towards one another and social indifference or even aggression towards novel individuals. This phenomenon is measurable in the laboratory via the partner preference test: a test animal is allowed to interact with a familiar and a stranger conspecific in a three-chambered arena, and the time spent in proximity to the familiar versus the stranger is quantified [12–16]. Also unlike laboratory rats and mice, prairie voles often choose social exploration over novel environment exploration [17]. Thus, prairie voles are uniquely suited to enable dissection of the nuanced behavioural aspects of social bonding, many of which may be relevant to aspects of human neurodevelopmental disorders, including ASD.

Normally, prairie voles require between 6 and 72 h of cohabitation with an opposite sex mate to form a pair bond—the duration of which may vary depending on several reported factors, including sex, various stressors, hormonal status and presence of mating [16,18–20]. Recently, we have shown that early-life sleep disruption (ELSD) also affects the expression of pair bonding in adult prairie voles after cohabitation [12]. Specifically, we exposed prairie vole pups to ELSD during the third postnatal week. Once animals reached adulthood (approx. 100 days old), the male animals showed reduced affiliative interactions with their female partners during the post-cohabitation partner preference test [12]. Intriguingly, social memory and selective aggression towards the stranger were both preserved in the partner preference test, suggesting that the effects of ELSD are specific to affiliative interactions [12]. However, our understanding of the effects of ELSD on affiliative behaviour has been limited by two factors: (i) manual scoring of social behaviours such as mating, affiliation or aggression; and (ii) the partner preference test examines the short-term *expression*, but not the long-term *formation* of the pair bond. Addressing these limitations requires automated, long-term recording of ELSD prairie vole cohabitation devoid of behavioural scoring. Without this type of recording, we may miss behavioural signatures of pair bond formation, including ELSD-related alterations. In fact, many aspects of social behaviours in humans, including interest in other conspecific individuals, also vary continuously over prolonged periods, including subtle alterations in energy, motivation or sleep due to pathological states. This links closely with the 6–72 h monitoring of ELSD prairie vole cohabitation. Thus, automated analysis of prairie vole cohabitation can provide new insights into social bonding and its alterations.

Automated analysis of animal behaviour involves moving away from manual video scoring and its subjectivity [21]*.* Whereas manual scoring relies on a fixed set of rules (e.g. proximity and stillness of animals) to discretize periods *containing* certain behaviours (e.g. huddling), automated methods can derive features of animals across time and space [22], enabling temporally and spatially continuous measures without prior assumptions. Using such computational tools to better study animal behaviour and its neural underpinnings make up the field of computational neuroethology [23]. One such tool is ‘deep learning’, which involves artificial neural networks capable of learning features of raw data and making predictions based on such learning [24,25]. Programs that apply deep learning for video analysis (e.g. DeepLabCut, SLEAP, DeepEthogram) have enabled markerless tracking of body location and posture in a diverse set of vertebrates and invertebrates: a technique known as ‘pose estimation’ [26–28]. In prairie voles, pose estimation has lagged compared with other rodent species, probably because of technical challenges imposed by affiliative behaviours that are typical of prairie voles (e.g. huddling). Some progress is being made to address these pose-estimation challenges, including previous work in prairie voles demonstrating that the centroid of a focal animal can be tracked across time in a three-chamber assay of partner preference using idTracker.ai [29,30] and unpublished work demonstrating the efficacy of a random forest model to classify social behaviour (courtesy of Aakriti Lakshmanan, laboratory of Dev Manoli, [31]). However, none of this work has enabled measurement of the process itself leading to a pair bond. We just have modest information from a few prior studies, including our own unpublished work, showing that pair bonding still occurs in voles in the absence of mating, so long as the cohabitation period is adequate (at least 24 h in females [16,32], at least 72 h in males—unpublished). Thus, pose estimation remains underused in prairie voles, and we are still far from realizing the full potential of pose estimation for the quantification of subtle social behaviours, such as the process of pair bonding and its sleep-related alterations.

In order to address this gap, herein we report pose-estimation data characterizing subtle behavioural alterations during the time course of pair bond formation in prairie voles exposed to ELSD. We used a 72 hour cohabitation protocol with a male and female prairie vole pair separated by a mesh divider. We then applied deep learning followed by custom analyses to identify subtle changes in social behaviour across the time span of pair bond formation. Because of the length of our video recordings, we were also able to capture temporal patterns of behaviour at the timescale of hours. Two strategies were used to achieve these novel kinds of data: (i) overhead video at 90° angle, so that male and female individuals could be tracked in their respective two-dimensional spaces without spatial overlap and with reproducibility across recordings, generating well-controlled measures of within-pair interactions; (ii) continuous recording across the 72 h period with enough temporal resolution to capture social dynamics, such as the latency with which the animals habituate to each other, as well as the incidence of social interactions over time, as measured by body orientation towards the conspecific. Additionally, ultradian periodicity (cycles in the scale of hours) has been previously seen to be a significant modulator of prairie vole behaviour in field studies [33], and thus we determined whether this rhythmicity might be altered in ELSD voles. Our investigation has resulted in the confirmation of prior findings that ELSD males show more profoundly impaired affiliative orientation [12], as well as alterations in ultradian rhythmicity of social behaviour. With our approach, we also observed new, subtle social phenotypes across both sexes with regard to both the timing and spatial topography of within-pair interactions.

## 2. Methods

### 2.1. Subjects

**Figure 1.**
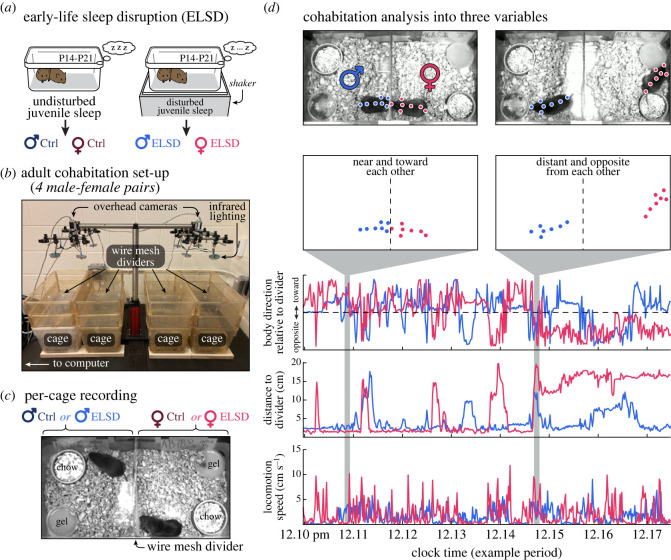
Juvenile sleep disruption and adult video recording. (*a*) Prairie vole litters were exposed to gentle cage agitation during a sensitive developmental window (P14–P21), resulting in ELSD [12]. (*b,c*) Once adults, ELSD animals (or their controls) were assigned to mutually naive male–female pairs. These pairs were monitored from an overhead angle for 72 h under circadian light/dark switching. Each camera recorded two divided cages, for a total of four animals per camera, i.e. two male–female pairs. Opaque barriers were placed between cages, preventing the pairs from distracting each other. (*d*) Using DeepLabCut [26] on video from each animal individually, animals were tracked as if they were moving arrows, before being recombined into pairs. Behavioural tracking data were then processed into standardized measures across recordings: body direction, distance from divider and locomotion speed. Body direction angles were normalized in the −1 (opposite) to +1 (toward) scale, i.e. clockwise and counterclockwise body rotations were treated equally in this analysis.

Prairie voles were bred and reared by both parents at the Veterans Affairs Portland Health Care System. Litters with both males and females were submitted to early-life sleep disruption (ELSD) or control conditions when pups were at postnatal day 14–21 (P14–P21; figure 1*a*; see below for ELSD procedure). Subjects were weaned at P21 into groups of two–four same-sex siblings per cage and co-housed at the same breeding site until reaching adulthood. The groups of siblings were transferred between P50 and P90 to the University of Michigan Medical School for the main recordings. Prairie voles were allowed to acclimatize to the facility transfer for two weeks prior to experimentation. Housing conditions were the same throughout experiments, including controlled temperature (20–23°C), ventilation (double-filtered outside air, negative pressure), humidity (30–70%), bedding (rolled paper pellets), ad libitum food (mixed diet of rabbit chow, corn and cracked oats), ad libitum water (bottles/hydrogel), environmental enrichment (cotton nestlets and wooden blocks/sticks), and 14 : 10 h light/dark cycle (lights on at 05.00). Cages and nestlets were changed weekly. The prairie voles we used derived from a colony at Emory University (Dr Larry Young), which in turn originated from field-caught animals in Illinois. Genetic diversity has been maintained through bi-annual donations among researchers across the USA (North Carolina State, University of California Davis, University of Colorado Boulder and Florida State University).

### 2.2. Early-life sleep disruption

Litter-containing home cages with both parents were placed on an orbital shaker (turned on every 110 s for 10 s, 110 rotations per minute) when pups were at P14–P21 of age, thus generating ELSD phenotypes. Control animals were moved into the room with the shakers, but cages were not agitated (figure 1*a*). Hydrogel was provided instead of water bottles during orbital shaking to prevent spillage in ELSD cages. Hydrogel was equivalently provided in control cages. As described by our previous study [12], ELSD is a gentle sleep disturbance method that predominantly affects infant sleep while preserving parental care and hormonal markers of stress. Specifically, our ELSD method was validated in separate studies using EEG recordings in juvenile prairie voles [12,34]. According to such data, ELSD disrupts sleep by decreasing REM sleep time by more than 20% per 24 h period. No such recordings were conducted in the present study. However, we used the same sleep disruption protocol following identical cage agitation parameters.

### 2.3. Cohabitation recording

Individuals emerging from the housing and sleep manipulations described above were submitted to cohabitation recordings during adulthood (26.5 ± 6.9 weeks of age, mean ± standard error). All adults were sexually naive, and each adult was assigned to an opposite-sex mate. Pairs were formed randomly with all possible sex versus sleep manipulation combinations: Male-ELSD/Female-ELSD (eight pairs), Male-ELSD/Female-Ctrl (six pairs), Male-Ctrl/Female-ELSD (seven pairs), Male-Ctrl/Female-Ctrl (seven pairs), for an initial total of 56 individuals. The resulting numbers of individuals by sex/ELSD groupings were as follows: Male-Ctrl (*n* = 13), Male-ELSD (*n* = 14), Female-Ctrl (*n* = 13), Female-ELSD (*n* = 15), for a final total of 55 individuals (one Male-Ctrl individual was excluded from analysis due to low locomotion activity throughout the recording). Male and female pairs were placed in bedded home cages (48.3 cm length, 25.4 cm width, 20.3 cm height), with individuals separated from each other by a laboratory-made mesh divider, meaning that individuals could only roam within the confinements of their assigned home cage areas (figure 1*b,c*). Cage dividers were made with metal wire mesh (square mesh, 6.5 mm aperture, 22 cm wide, 28 cm high) covered on both sides with plastic sheet (clear polycarbonate, 0.5 mm thick) to prevent animals from climbing. The bottom rectangular portion of the mesh barrier was left exposed without the plastic sheet (6.5 cm high), allowing animals to exchange bedding and sniff each other. Crocs with chow/gel were placed consistently across recordings, with chow and gel being always positioned away from the divider, on the left and right sides of the animal, respectively. No environmental enrichment objects were offered during recordings, thus forcing animals to interact with each other or the mesh divider. Finally, we placed cardboard barriers between neighbouring cages, preventing male–female pairs from distracting each other.

Animals were allowed to behave freely while being filmed from a 90° overhead angle for 72 h, starting always at 08.00 (i.e. 3 h after lights on). For video recording, we customized a vibration-free optomechanical assembly (ThorLabs) holding two infrared-sensitive cameras (specifications below), each camera surrounded by four infrared illuminators (made in the laboratory from inexpensive LED boards). Two mesh-divided home cages were placed below each camera, allowing to record four male–female pairs at once (figure 1*b,c*). This system was installed in a housing-approved room with circadian light/dark switching. Light/dark switching was innocuous to video brightness, as imaging was obtained with infrared reflectance.

We used two greyscale cameras (Basler, acA1300–60gm), each one attached to a fixed focal length lens (Edmund Optics, 6 mm UC Series). The cameras communicated via a network adapter (Intel Pro 1000/PT) with a host computer, and videos were written into an array of hard disks (RAID) for protected data storage. Cameras were configured in Pylon software (Basler) with 8-bit depth, 800-pixel frame width, 896-pixel frame height (no binning), 1328 kbps and 20 Hz frame rate. Exposure and brightness were adjusted by manipulating the lenses and infrared illuminators, without further adjustments to the camera software. Videos were acquired using StreamPix software (NorPix) into 6 h mp4 segments (H.264 codec) from the two cameras synchronously. Each camera recorded two male–female pairs, i.e. four video quadrants. Quadrants were reframed using Adobe Premiere and re-exported using Adobe Media Encoder, resulting in smaller videos with one individual per video (384-pixel frame width, 416-pixel frame height, 1025 kbps, 25 Hz frame rate, mp4 format, H.264 codec).

### 2.4. Behavioural tracking

Reframed videos were submitted to markerless body tracking using DeepLabCut (DLC) v. 2.2 [26]. We opted for DLC because it is free open-source software and because of its raw output data (in pixel coordinates), which allowed us to fully customize our own data analysis code (see Data analysis below). We trained the DLC network (*resnet_50*) to label seven body parts per individual: nose, left/right ears, shoulder, two locations along the back and tail base (figure 1*d*). For network training, we used manually selected video frames (*n* = 157) representing a variety of scenarios: from clear imaging of the animal (no motion blur or obstruction of body parts) to challenging situations (e.g. with motion blur, curled posture when sleeping or eating, tail base hidden under bedding, nose hidden by the mesh divider when sniffing the cage mate) across recordings from representative animals, three males and three females. We then trained the network overnight using a laboratory server (operating system: CentOS, a Linux distribution. CPU: Intel Xeon E5-2640 v. 3 @ 2.60 GHz. RAM memory: 512 GB. GPU: NVIDIA GeForce GTX 1080 Ti). After network training, we extracted pixel coordinate outputs from DLC, along with human verification of quality via inspection of analyses provided by DLC itself, like trajectory plots. Approved recordings were then submitted to the custom analyses described next. See also electronic supplementary material, movie S1.

### 2.5. Data analysis

Body part coordinates per video frame were saved as CSV files, imported to Matlab (MathWorks) and converted from pixels to centimetres. We then obtained three measures per video frame (figure 1*d*) explained as follows. (i) Body direction relative to the divider: we fitted a line to the sequence of coordinates from tail to nose and determined the angle of the fitted line relative to the horizontal plane of the video frame. The angle was then rescaled from −1 (opposite from divider) to +1 (toward divider), with left and right directions treated equally. By doing this we eliminated the circular dimension, i.e. we ignored information on clockwise or counterclockwise body rotations, which were outside of our scope. This resulted in a straightforward measure of ‘interest’ in the partner vole through the mesh divider. (ii) Distance to divider: length between shoulder and divider on the horizontal axis of the video frame, regardless of the position on the vertical axis. In the absence of unrestrained touch between animals, this measure provided a measure of proximity. (iii) Locomotion speed: difference (hypotenuse) between the shoulder coordinates of frame *n* and frame *n* + 1. Prior to further analysis, the accuracy of body direction, distance to divider, and locomotion speed measures was manually checked by inspecting short video segments per male–female pair along with their corresponding data, as illustrated in electronic supplementary material, movie S1.

All measures were time-stamped per video frame according to clock time in number of seconds × number of frames per second. For example, the first frame after 08.00 on Day 1 of recording was identified as 720 001 (8 h × 60 min × 60 s × 25 frames + 1 frame). Time-stamping was made without restarting the clock at midnight, so that each frame could have a unique identifier across the 72 h recording. These time-stamping procedures were used to align all recordings onto a common 72 h axis, given that all recordings were intentionally made with 15–30 min margins for later trimming. Clock time information was obtained from the file naming system of the video acquisition software (StreamPix, NorPix).

**Figure 2.**
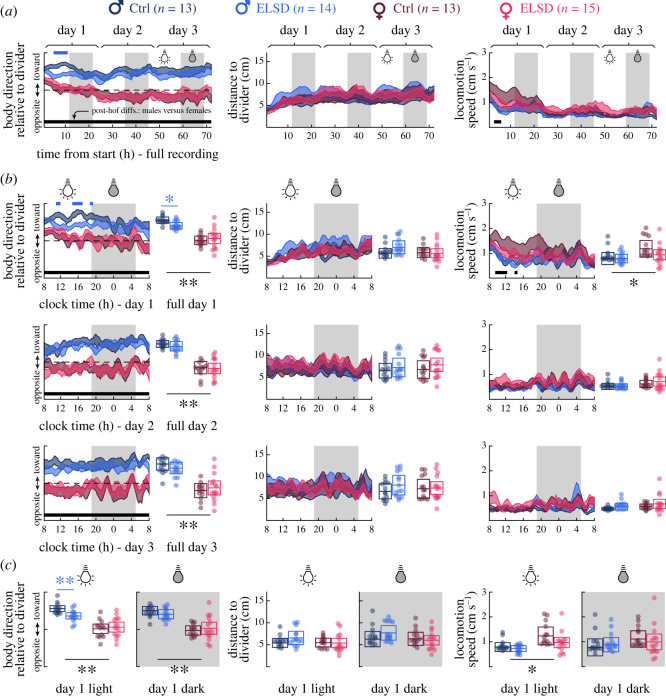
Male-ELSD prairie voles exhibit less orientation towards females in the initial 12 h of cohabitation. (*a*) Behavioural measures across 72 h cohabitation in 1 h bins. Light/dark periods are indicated with background shading. Shaded curves represent standard errors around means. Both male groups were more likely than females to orient toward the divider throughout the experiment (left graph) and both female groups showed higher locomotor activity than males in the initial hours of recording (right graph), as indicated by *post hoc* differences (black bars on bottom). All groups showed a tendency to evolve from high activity near the divider on Day 1 to lower activity in a broader home cage area in later periods. Male-ELSD animals were less likely than Male-Ctrl to orient toward the divider during the initial 12 h of recording (blue bar on top). *Post hoc* differences: *p* < 0.01. (*b*) Curve graphs: same data, but in 20 min bins and divided into days of recording. Male-ELSD animals were indeed less likely than Male-Ctrl to behave toward the divider. Box plots: averages from the corresponding curve graphs, reinforcing the difference between male groups (blue asterisks), as well as between-sex differences (black asterisks). (*c*) Averaged box plot data like (*b*) but focused on Day 1. The Male-ELSD effect of (*b*) coincided with the light period of Day 1. *p < 0.05. **p < 0.005.

Body direction relative to divider, distance to divider and locomotion speed data per individual were averaged into 1 h bins (figure 2*a* curves) or 20 min bins separated into the three recording days (figure 2*b* curves). In either case, binned data were first submitted to statistical comparisons between sexes (ELSD and Ctrl combined) to characterize sex-specific behavioural features in our divided-cage recordings. Then, we compared ELSD versus Ctrl treatments within each sex (two-way repeated measures ANOVA, followed by Tukey's *post hoc* comparisons at each time bin). This allowed us to differentiate basal sex specificities from ELSD-induced effects. The same data were averaged across 24 h periods (figure 2*b* box plots) or light/dark periods of Day 1 (figure 2*c* box plots) and submitted to one-way ANOVA, again with combined male versus female comparisons and—separately—ELSD versus Ctrl comparisons per sex.

**Figure 3.**
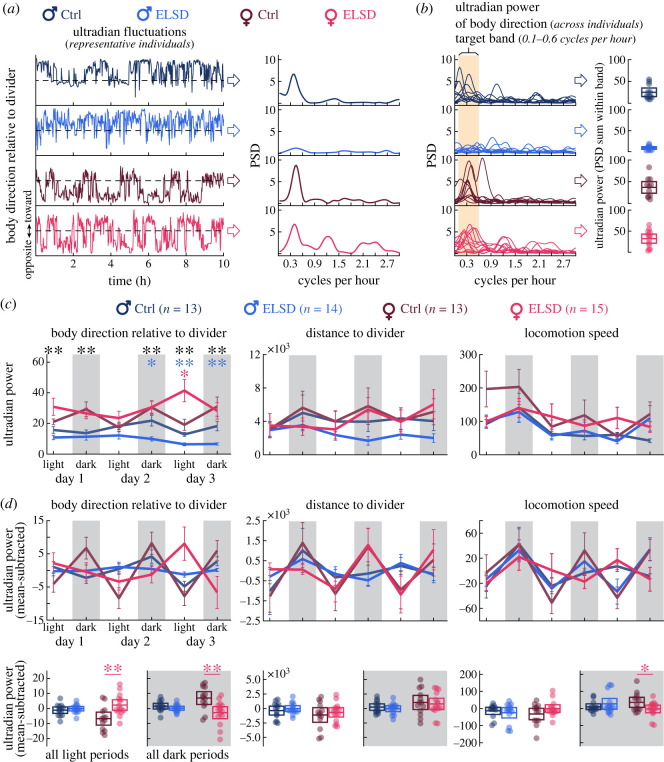
ELSD affects body direction rhythms in sex-specific manners. (*a*) Left: 10 h curves (1 s bins) from representative individuals per group, showing body direction fluctuations recurring every 2–3 h. Right: power spectral density (PSD) of such fluctuations, showing peaks at around 0.4 cycles h^−1^. The PSD peak was lower in the Male-ELSD individual. (*b*) Left curves: PSD curves for all animals in each group, showing that PSD peaks were indeed lower in Male-ELSD animals as a group. By summing PSD values within the frequency band containing most of PSD peaks (0.1–0.6 cycles h^−1^) we obtained band power values per individual, i.e. ‘ultradian power’ of body direction. Right box plots: ultradian power of body direction across groups. (*c*) The same analysis as in (*b*), but across behavioural measures and recording periods. Females showed stronger ultradian periodicity of body direction than males (black asterisks). Male-ELSD animals showed weaker ultradian periodicity in body direction than Male-Ctrl from Day 2 (blue asterisks). Female-Ctrl animals seemed to show a circadian fluctuation in the ultradian power of body direction, and this effect was not as apparent in Female-ELSD. We analysed this more deeply in (*d*). (*d*) Circadian fluctuations of ultradian power were magnified by mean-subtracting the data in (*c*). Light and dark periods were then separately averaged and depicted as box plots. This revealed a female-specific effect of ELSD: a disruption of both ultradian and circadian behavioural rhythms, especially in the body direction variable. *p < 0.05. **p < 0.005.

The three behavioural measures were also examined for ultradian periodicity (biological cycles in the scale of hours), an exploratory approach to evaluate if the above behaviours oscillate in an ultradian manner. Data were resampled from the original video frame rate to 1 s bins and analysed using Welch's power spectral density (PSD) estimate (6 h Hamming windows, frequency range of 0–3 cycles h^−1^ in steps of 0.03 cycle). By examining the PSD curves, we found that peaks were mostly prominent within the 0.1–0.6 cycle h^−1^ band, which we interpreted to represent the ultradian fluctuations we observed in the raw data (figure 3*a,b* curves). Thus, we summed PSD values within the 0.1–0.6 cycle h^−1^ band per individual (figure 3*b* box plots) and did the same across behavioural variables and time periods (figure 3*c*). Differences between sexes (ELSD and Ctrl combined) or between ELSD and Ctrl treatments per sex were examined using two-way repeated measures ANOVA, followed by Tukey's *post hoc* comparisons at each period, like the comparisons made in figure 2. We additionally subtracted ultradian power curves by their own moving averages with a sliding window of three data points (i.e. three periods) on a per-animal basis (figure 3*d* curves). This resulted in curves with magnified light/dark alternation, representing circadian fluctuations. Data from light and dark periods were then separately averaged (figure 3*d* box plots). Between-sex and within-sex comparisons per light or dark period were made using one-way ANOVA.

**Figure 4.**
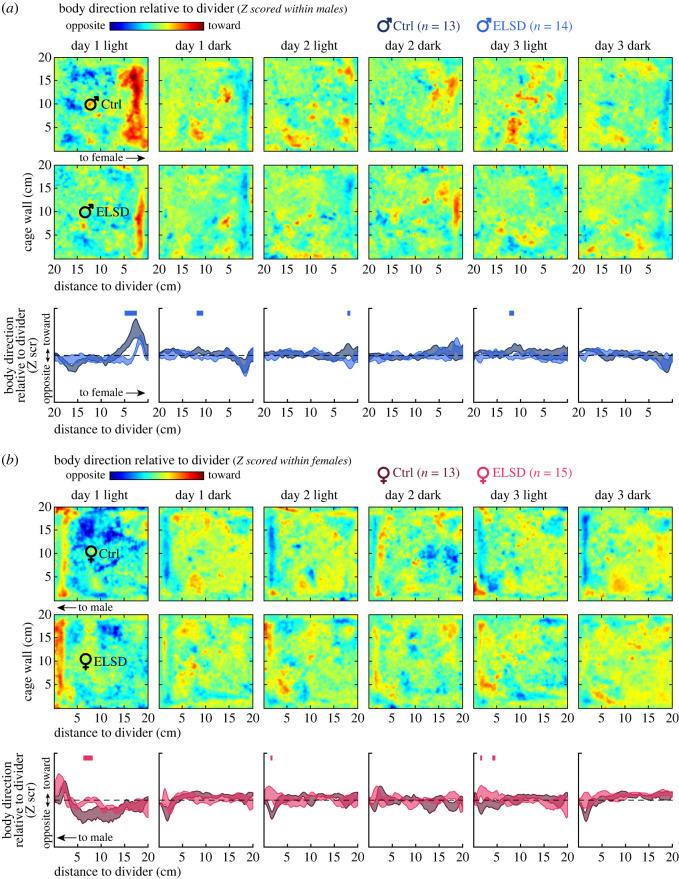
ELSD alters both spatial occupancy and body orientation in sex-specific manners. (*a*) Heatmaps: body direction was mapped onto square home-cage areas (spatial axes, 2 mm grids) per recording period, and Z-scored across recording periods. Images are averages across the animals of each male group. Male-Ctrl animals spent more time near and toward the divider, especially during the light period of Day 1. Curves: heatmaps from individual animals and recording periods were averaged vertically, generating body direction versus distance to divider curves. The standard errors in this panel represent variations from such curves. Male-ELSD animals were less likely to behave near and toward the divider on Day 1, light period. Blue bars on top indicate *post hoc* differences at distance-to-divider bins (*p* < 0.05). (*b*) Same analysis, but from females. Female-ELSD animals were less likely to point opposite from the divider while occupying the centre of the assigned home cage area, particularly during the light period of Day 1. Across periods, Female-ELSD animals were slightly more likely than Female-Ctrl to behave near and toward the divider.

Finally, using data in 1 s bins, we created spatial maps depicting both area occupancy and body direction (figure 4). A 100 × 100 cell array was created in Matlab to represent a 2 mm grid of the home cage area assigned to each animal (dimensions: 20 × 20 cm). The cell array was cumulatively populated with body direction values across time bins, according to the animal's position at each time bin. We then averaged the values per cell, which resulted in the maps. Six maps were created per individual, corresponding to the light/dark periods of Days 1–3. Such maps were arranged three-dimensionally and Z scored across the third dimension within males or females (represented by averages per group in the heatmaps of figure 4). We then averaged each map vertically to obtain body direction versus distance to divider curves. These curves were submitted to within-sex statistical comparisons (two-way repeated measures ANOVA, followed by Tukey's *post hoc* comparisons per spatial bin; figure 4 curves).

## 3. Results

### 3.1. Male-ELSD prairie voles exhibit less orientation towards females in the initial 12 h of cohabitation

Figure 2*a* shows behavioural measures in 1 h bins, across the 72 h cohabitation. First, we compared males versus females irrespective of ELSD or Ctrl treatments. Across time bins, we observed that males were constantly more likely to behave toward the divider (figure 2*a*, left graph) (effect of sex grouping: *F*_1,3763_ = 2.8 × 10^3^, *p* < 0.0001; group versus time interaction: *F*_71,3763_ = 1.538, *p* = 0.0027; electronic supplementary material, table S1, rows 1 and 3) and females showed higher locomotion speed, particularly in the initial hours of recording (figure 2*a*, right graph) (effect of sex grouping: *F*_1, 3763_ = 162.625, *p* < 0.0001; electronic supplementary material, table S1, row 19). This suggests that males spent more time investigating the partner, whereas females were more likely to roam across the home cage area, especially earlier during cohabitation. Also, across sexes, we observed a gradual increase in distance to divider (figure 2*a*, centre graph) (effect of time: *F*_71, 3763_ = 3.416, *p* < 0.0001; electronic supplementary material, table S1, row 11) and gradual decrease in locomotion speed (figure 2*a*, right graph) (effect of time: *F*_71, 3763_ = 10.387, *p* < 0.0001; electronic supplementary material, table S1, row 20). This suggests a transition from higher activity near the divider on Day 1 (possibly due to novelty) to lower activity in a broader home cage area in later periods (possibly due to habituation).

Then, we examined ELSD versus Ctrl animals per sex. During the initial 12 h of recording, Male-ELSD animals were less likely than Male-Ctrl to orient toward the divider (figure 2*a*, left) (effect of Male-ELSD versus Male-Ctrl grouping: *F*_1,1775_ = 62.188, *p* < 0.0001; electronic supplementary material, table S1, row 4) and near the divider (figure 2*a*, centre) (effect of Male-ELSD versus Male-Ctrl grouping: *F*_1,1775_ = 41.083, *p* < 0.0001; electronic supplementary material, table S1, row 13), though this latter effect was not accompanied by *post hoc* *p* values lower than 0.05 across the time bins. To further specify these differences, we performed ELSD versus Ctrl comparisons at higher temporal resolution (20 min bins) and in separate 24 h segments aligned with clock time, as shown in figure 2*b*. When analysing Day 1 alone, we found stronger differences between Male-ELSD and Male-Ctrl in terms of body direction (figure 2*b*, top left curve graph) (effect of Male-ELSD versus Male-Ctrl grouping: *F*_1,1775_ = 121.205, *p* < 0.0001; group versus time interaction: *F*_71,1775_ = 1.397, *p* = 0.0176; electronic supplementary material, table S2, rows 4 and 6) and distance to divider (figure 2*b*, top centre curve graph) (effect of Male-ELSD versus Male-Ctrl grouping: *F*_1,1775_ = 87.486, *p* < 0.0001; electronic supplementary material, table S2, row 13; no *post hoc* *p* values lower than 0.05), again suggesting higher activity near and toward the divider in Male-Ctrl animals. We additionally observed that Female-Ctrl animals showed higher locomotion speed than Female-ELSD (figure 2*b*, top right curve graph) (effect of Female-ELSD versus Female-Ctrl grouping: *F*_1,1846_ = 61.975, *p* < 0.0001; electronic supplementary material, table S2, row 25; no *post hoc* *p* values lower than 0.05). We also averaged across the time bins of Day 1 per animal and again found a significant body direction difference between the two male groups (figure 2*b*, top left box plots; *F*_1,25_ = 6.050, *p* = 0.0212; electronic supplementary material, table S3, row 4). Differences between sexes irrespective of ELSD or Ctrl treatments during Day 1 were again present in body direction curves (effect of sex grouping: *F*_1,3763_ = 1.5 × 10^3^, *p* < 0.0001; electronic supplementary material, table S2, row 1) and box plots (F_1,53_ = 57.257, *p* < 0.0001; electronic supplementary material, table S3, row 1) (figure 2*b*, top left), as well as locomotion speed curves (effect of sex grouping: *F*_1,3763_ = 162.716, *p* < 0.0001; electronic supplementary material, table S2, row 19) and box plots (*F*_1,53_ = 4.644, *p* = 0.0357; electronic supplementary material, table S3, row 19) (figure 2*b*, top right). This confirms the sex-specific behaviours during Day 1, i.e. slower activity toward the partner in males—especially Male-Ctrl—and faster activity across the home cage in females—especially Female-Ctrl.

Figure 2*c* focuses further on Day 1 by separately averaging its light and dark periods. During the light phase, there was an even stronger difference between the male groups in terms of body direction (figure 2*c*, left) (*F*_1,25_ = 10.975, *p* = 0.0028; electronic supplementary material, table S3, row 5) and between sexes in terms of locomotion speed (figure 2*c*, right) (*F*_1,53_ = 8.285; *p* = 0.0058; electronic supplementary material, table S3, row 20). These differences coincide with the period of novelty-induced home-cage activity explained above, suggesting a relationship between environmental novelty and altered Male-ELSD behaviour (circadian influences not necessarily involved; see Discussion).

Therefore, figure 2 demonstrates an optimal time interval and behavioural measure to capture ELSD effects on male prairie voles, in addition to demonstrating sex differences in body direction and locomotion speed irrespective of ELSD treatment. These findings suggest that ELSD preferentially impairs the social components of male behaviour in the initial hours of cohabitation.

### 3.2. Early-life sleep disruption affects body direction rhythms in sex-specific manners

Interestingly, the curve plots in figure 2*b* also show somewhat regular fluctuations across all behavioural measures and groups of animals. These fluctuations suggest cycles in the timescale of around 2–3 h, i.e. ultradian rhythms, especially during dark periods. We address this in figure 3.

Figure 3*a* shows fluctuations in body direction over a 10 h period for one representative individual per group of animals. It also shows the power spectral density (PSD) of those fluctuations, and which frequencies were most prevalent. PSD peaked at around 0.4 cycles h^−1^, with strong peaks in all individuals except the Male-ELSD one. Figure 3*b* exhibits PSD curves from all individuals per group, illustrating that PSD peaks appeared indeed weaker in Male-ELSD animals. We quantified this by summing the 0.1–0.6 cycle h^−1^ PSD values per individual, as exemplified in the box plots of figure 3*b*. Here we will refer to 0.1–0.6 cycle h^−1^ as the ‘ultradian band’, corresponding to cycles of behavioural activity recurring every 2–3 h approximately.

The ultradian power analysis exemplified in figure 3*b* is from body direction during the dark period of Day 2. The same analysis is shown in figure 3*c*, but across behavioural variables and time periods. Particularly in the body direction variable, females were more likely than males to exhibit ultradian periodicity throughout the 72 h cohabitation, irrespective of ELSD or Ctrl status (figure 3*c*, black asterisks) (effect of sex grouping: *F*_1,265_ = 84.807, *p* < 0.0001; electronic supplementary material, table S4, row 1). This indicates that females are more likely than males to reorient themselves every 2–3 h. Within-sex effects of ELSD on body direction were confined to later cohabitation periods: Male-ELSD showed constantly weaker ultradian power, especially starting on Day 2 (effect of Male-ELSD versus Male-Ctrl grouping: *F*_1,125_ = 35.740, *p* < 0.0001; electronic supplementary material, table S4, row 4). This indicates that ELSD further reduces the tendency of males to reorient themselves every 2–3 h, especially as they habituate to the environment.

Figure 3*c* also shows a female-specific effect of ELSD on body direction. First, we noticed that Female-Ctrl animals show a fluctuation in ultradian power across periods: weaker in light, stronger in dark. We then noticed a disruption of such pattern in Female-ELSD, including a localized increase in ultradian power during the light period of Day 3 (effect of Female-ELSD versus Female-Ctrl grouping: *F*_1,130_ = 3.925, *p* = 0.0493; electronic supplementary material, table S4, row 7). This suggested a sex-specific and ELSD-sensitive relationship between the ultradian and circadian timescales, which we investigated more deeply in figure 3*d*. Ultradian power curves of each animal were subtracted by their own moving averages. This resulted in the detrended curves of figure 3*d*, with clearer light/dark cycling. We then averaged light and dark periods separately per animal, producing the box plots of figure 3*d*. The strongest effects were found in the ultradian power of body direction. More specifically, Female-Ctrl animals were observed to alternate between lower and higher ultradian power during light and dark periods, respectively. This pattern was disrupted in Female-ELSD (figure 3*d*, bottom left) (light period: *F*_1,26_ = 10.265, *p* = 0.0036; dark period: *F*_1,26_ = 10.588, *p* = 0.0032; electronic supplementary material, table S5, rows 5 and 6). Female-ELSD additionally showed a slightly lower ultradian power of locomotion speed during dark periods than controls (figure 3*d*, bottom right) (*F*_1,26_ = 4.327, *p* = 0.0475; electronic supplementary material, table S5, row 18).

Electronic supplementary material, table S4 shows additional statistical outputs regarding the distance to divider and locomotion speed curves of figure 3*c*, none of which accompanied by *post hoc* *p* values lower than 0.05. The only additional within-sex difference was observed in the distance to divider curves from Male-ELSD and Male-Ctrl (figure 3*c*, centre) (*F*_1,125_ = 6.872, *p* = 0.0097; electronic supplementary material, table S4, row 13). Light versus dark comparisons in distance to divider and locomotion speed showed no statistical effects, except for the relatively weak effect in female locomotion speed reported above (figure 3*d*). This suggests that ELSD preferentially impairs body direction rhythms in sex-specific manners, while sparing more general aspects of home cage roaming, consistent with figure 2. Finally, it should be noted that ultradian periodicity was evident even after averaging across animals, indicating that not only are rhythms present, but the same rhythms may be present across individuals. This suggests that individual animals' rhythms are in phase with one another across different times of day.

### 3.3. Early-life sleep disruption alters both spatial occupancy and body orientation in sex-specific manners

We next mapped body direction data onto the cohabitation cage area, resulting in the spatial patterns of figure 4 (2 mm grids). The heatmaps in figure 4*a* were produced by Z scoring across male animals and recording periods, and then separately averaging across Male-Ctrl or Male-ELSD animals per recording period. During the light period of Day 1, we observed an area near and along the divider where animals spent more time orienting toward the divider, especially Male-Ctrl animals (figure 4*a*, leftmost heatmaps; see red areas within 5 cm from the divider). To quantify this, the heatmap of each individual animal was averaged vertically into a body direction versus distance to divider curve. Standard errors from such curves are presented as the bottom graphs of figure 4*a*. During the light period of Day 1, we observed that Male-Ctrl animals indeed spent more time near and toward the divider (figure 4*a*, leftmost curve graph) (effect of Male-ELSD versus Male-Ctrl grouping: *F*_1,2475_ = 75.905, *p* < 0.0001; group versus distance interaction: *F*_99,2475_ = 2.429, *p* < 0.0001; electronic supplementary material, table S6, rows 1 and 3). With this, we clarify the spatial pattern behind the body direction results of figure 2.

Figure 4*b* shows the same subplot layout and analysis, but from females. The stronger differences were again observed during the light period of Day 1. However, ELSD effects on female body direction were observed at a wider and more central location in the cage, 5–15 cm from the divider. Interestingly, at this location and day period, Female-Ctrl animals spent more time facing opposite from the divider than Female-ELSD animals (figure 4*b*, leftmost curve graph) (effect of Female-ELSD versus Female-Ctrl grouping: *F*_1,2574_ = 21.837, *p* < 0.0001; electronic supplementary material, table S7, row 1). No such effect had been observed in males (compare with figure 4*a*, leftmost plots). Thus, during the initial period of Day 1, here considered as the ‘novelty’ period (figures 2 and 3), higher behavioural activity enabled sex-specific body direction patterns across the home cage. These patterns could only be revealed due to the spatial mapping and Z scoring used in figure 4, complementing figures 2 and 3. We interpret these results as possibly sex-specific courtship displays, intermittent partner evaluation behaviours, or area preference patterns that are susceptible to ELSD. See also electronic supplementary material, movie S1.

Later recording periods in figure 4 showed smaller and spatially localized differences between ELSD and Ctrl per sex, as reported in electronic supplementary material, tables S6 and S7. Particularly, when compared with Female-ELSD, Female-Ctrl animals were slightly more likely to orient themselves away from the divider when near it (figure 4*b*; see narrow blue areas within 5 cm from the divider). This could reflect a behaviour we sometimes observed during quiet wake or sleep periods, when animals rested their bodies against the divider, with their heads pointing slightly away from the divider, i.e. diagonally. We interpreted this behaviour as possibly motivated by attempts to huddle with the other animal through the mesh divider, as this behaviour was not regularly observed with any of the other three walls of the enclosure, which did not have a social stimulus on the other side. The difference between female groups in this putative huddling-like behaviour was subtle and specific to the light period of Day 2 (group versus distance interaction: *F*_99,2574_ = 1.635, *p* < 0.0001; electronic supplementary material, table S7, row 9), when Female-ELSD animals tended to rather point toward the divider when near it. These late-recording results may shed light on both the sex specificity and ELSD sensitivity of subtle prairie vole behaviours, though these effects were generally much weaker than those of the initial period of Day 1 and are therefore interpreted cautiously here.

## 4. Discussion

Our previous work indicated that early-life sleep disruption (ELSD) impaired social behaviour in prairie voles, which we had only assessed during a limited three-hour partner preference test: a laboratory-based ‘readout’ of pair bonding *after* the bond is formed [12]. In the present study, we extend our previous findings by using automated pose estimation of opposite-sex ELSD prairie vole pairs *during* a 72 h cohabitation period—the process leading up to partner preference behaviour and therefore pair bond formation. Our findings generated novel observations about alterations in more nuanced spatio-temporal aspects of social behaviour induced by ELSD. Thus, by applying deep learning approaches to examination of animal pose estimates, we both confirm and extend our previous male-specific findings in ELSD, and also discover new effects of ELSD on female vole social behaviour.

This work also highlights a broader cross-species phenomenon: early-life sleep is fundamental to the maturation of social behaviours. In fact, developmental sleep problems in humans are associated with social difficulties later in life, and socio-emotional processing depends on adequate sleep [35,36]. In addition, subtle social behaviours in humans, including impaired social entrainment in dyadic interactions in individuals with ASD, impose challenges to preclinical researchers who wish to model these behaviours [3]. Our experimental work in prairie voles is, therefore, informative for bridging the gap to translational research in humans.

Our laboratory has previously shown that male prairie voles subjected to ELSD exhibit impaired pair bond expression during a three-hour partner preference test as adults [12]. Early-life sleep disruption has also been experimentally performed in several other rodent species (see review, [6]), and has also shown a profound impact on later social behaviour. For example, using an identical ELSD protocol to ours in mice with *Shank3* (an ASD risk-gene), Lord and colleagues found that ELSD induces male-specific impaired sociality in adulthood [37]. In addition, automated sleep disruption from P14 to P21 or pharmacological reduction of REM sleep from P8 to P21 in rats was found to impact adult male sexual behaviour, increasing the time to mount and reducing the probability of ejaculation [38,39]. Finally, sleep disruption from P35 to P42 was observed to reduce sociality in adult mice, particularly their species-typical behaviour of social novelty preference [40].

Here, in alignment with our prior findings in ELSD males, we report that while all males generally show more orientation towards the partner than females in the first 12 h of cohabitation, ELSD males show less of this than control males (though still more than females). Thus, our findings in prairie voles are consistent with other broad social impairments described in other rodent species, but we are able to add detail as well as discover new patterns, as discussed below.

### 4.1. Early-life sleep disruption affects spatial area occupancy patterns upon first meeting

Our body direction and area occupancy analyses revealed stereotyped—and sex-specific—locomotor patterns not previously described in prairie voles: the behaviour of orienting and reorienting the body towards the partner, especially during the first 12 h of cohabitation. Our findings build further upon a computational neuroethology study that previously only tracked the centroid of interacting voles [30]. Here we added spatial and directional information to those interactions, resulting in sex-specific body orientation behaviours. These behaviours could be cautiously interpreted as a stereotyped ‘social dance’ of sorts—comparable to the one reported in other species, such as in *Drosophila* courtship behaviour [41,42]. This behaviour could also be the response to the mesh barrier interfering with typical prairie vole courtship behaviour [18,43,44]. Regardless of the potential confounds, these sex-specific spatial patterns were still significantly blunted in ELSD animals. This speaks to the ability of detecting nuanced behavioural changes when using a data-driven approach to ELSD prairie vole behaviour. However, it is unclear whether this locomotor pattern would still be observed in response to a same-sex conspecific, or a novel environment *without* a social stimulus. This is a potentially interesting future research direction to investigate. In addition, the strong spatial patterns observed in the initial 12 h did not persist over the subsequent 60 h of cohabitation. It is still unknown whether this temporal effect is due to the light phase in the first 12 h, or if this effect would still remain if cohabitation was started in the dark phase, which is another relevant question for future research.

### 4.2. Early-life sleep disruption affects behavioural rhythms across the 72 hour cohabitation

Circadian rhythms are influenced by social interactions and vice versa, and disruption of these rhythms in early life can have negative consequence on later social behaviour [45,46]. In the field, prairie voles are a crepuscular species, meaning they show ultradian patterns of heightened activity at dawn and dusk, which could be an adaptive strategy to avoid predation [47]. In our laboratory-based recordings, we also observed ultradian patterns of heightened locomotor activity, though these occurred every 2–3 h and were not entirely consistent with a naturalistic crepuscular pattern. Furthermore, we observed that ELSD affects these ultradian patterns of locomotor activity in complex manners, depending on both sex and light/dark periods.

Similar ultradian and circadian activity patterns have recently been reported in prairie voles housed in a semi-natural enclosure: a 0.4 hectare field from which telemetry signals were used to examine periodical patterns of locomotor activity [33]. In addition, an older study described ultradian and circadian periodicity of wheel running in the common vole (*Microtus arvalis*) [48]. This indicates that the temporal organization of vole behaviour—and probably of mammals in general—is robust enough to emerge in various experimental conditions, from the field to the laboratory. That was also the case in our home cage study, where behavioural activity was observed to be organized in cycles of 2–3 h. Remarkably, the same ultradian periodicity was evident across animals, suggesting that individual animals' rhythms were in phase with one another across different times of day. Thus, our results now demonstrate that ultradian cycles are not only present in gross behavioural measures such as locomotion, but also in dissected behavioural components, such as body direction relative to the cage mate. Crucially, it is unlikely that the ultradian periodicity we found in body direction was merely a by-product of general sex-unspecific behavioural rhythms, because ELSD effects were almost specific to the periodicity of body direction, in addition to being very different between males and females. This suggests that behavioural components may be orchestrated by separate ‘clocks’, each one involving sex-specific mechanisms with different susceptibilities to disturbances, such as ELSD. This notion is consistent with the common vole study mentioned above [48], according to which wheel running, as well as feeding behaviour, lose ultradian periodicity upon lesioning certain hypothalamic nuclei. Despite all of these findings, the purpose or predictive value of these fluctuations is unknown. Therefore, future vole studies involving manipulation of brain substrates relevant to social behaviours, such as the medial prefrontal cortex and its downstream circuits [49–51], could be highly informative if combined in real time with behavioural tracking applications, like the ones described here, as well as physiological measures (e.g. cardiac activity and body temperature), which are also known to fluctuate in ultradian rhythms in voles [52,53]. Even simple correlational studies between such fluctuating behaviours and subsequent pair bonding may lead to novel insights or biomarkers for bonding.

### 4.3. Limitations

There are several limitations in this study, in addition to the caveats mentioned above. First, in our experimental design in which cohabitation of animals took place using a mesh divider, a major limitation is that this set-up does not allow for assessment of true huddling, mating or other fully interactive social behaviours. Now that our protocol has been successfully established, we could add further complexity by allowing more naturalistic interactions in future studies. Despite this caveat, the divided-cage design remains useful for future studies involving chronic implants for brain electrophysiology, as the divider prevents animals from chewing each other's headcaps and/or recording cables.

Secondly, we did not test partner preference using traditional approaches in these animals. We have already shown a robust effect of ELSD on partner preference behaviour in previous work [12]. However, in the absence of partner preference data in the present study, we cannot infer what these cohabitation behaviours might mean for pair bond expression at a later time, nor can we make definitive conclusions regarding the pair bond status of these animals. Once we are able to allow further complexity and naturally interactive behaviours, it would be of great interest to apply our pose-estimation protocol to the partner preference test itself.

It also remains unclear whether the behavioural rhythms that we observe are driven by the light phase, and/or by sleep–wake cycles, and/or by circadian processes. Dissecting these potential confounders is beyond the scope of the current study, but should be studied in the future. Furthermore, here we did not sort our data into wake or sleep periods. Future studies with combined video and physiology monitoring should be able to make these distinctions, in order to quantify the ‘social variants’ of each state, e.g. mutual sniffing during active wake and huddling during sleep.

Finally, here we did not quantify prairie vole behaviour as a function of the ELSD/Ctrl status of the opposite-sex mate. For example, would an ELSD male behave the same when paired with an ELSD female versus a control female? Approaching this kind of interrogation would involve additional analyses not performed here (e.g. the probability of a certain male behaviour given the occurrence of a certain female behaviour), in addition to potentially requiring a higher number of male–female pairs for sufficient statistical power. These relational analyses were outside of our current scope but are certainly relevant for our future studies on ELSD.

## 5. Conclusion

Early-life sleep disruption has been shown to have long-lasting effects on social behaviour in adult prairie voles (*Microtus ochrogaster*), including impaired pair bonding with opposite sex mates during the conventional partner preference test. In this study, we used pose estimation to identify and quantify novel, more nuanced social behaviours, including temporally distinct locomotor patterns of approach and orientation towards the partner, as well as ultradian social activity rhythms. ELSD males and females showed significant blunting of these behaviours compared with control animals in a temporally distinct manner across the 72 h cohabitation period. Our findings highlight the utility of combining conventional manual behaviour tracking together with pose-estimation approaches in order to paint a comprehensive picture of social behaviour in animal models. In particular, this highly quantitative and temporally precise computer-based approach also opens doors to future time- and behaviour-based mechanistic studies aimed at either brain–behaviour correlations or intervention-based studies. Ultimately these more sophisticated approaches will aid in closing the gap in translation from preclinical to clinical studies, with the goal of understanding early-life sleep effects on heterogeneous neurodevelopmental disorders such as autism.

## Data Availability

Pre-processed data are available as a repository in Dryad (https://doi.org/10.5061/dryad.15dv41p1n) [54]. Statistical results are provided in electronic supplementary material [55].

## References

[1] Buckley AW, Rodriguez AJ, Jennison K, Buckley J, Thurm A, Sato S, Swedo S. 2010 Rapid eye movement sleep percentage in children with autism compared with children with developmental delay and typical development. Arch. Pediatr. Adolesc. Med. **164**, 1032-1037. (10.1001/archpediatrics.2010.202) 21041596 PMC3111973

[2] Cohen S, Conduit R, Lockley SW, Rajaratnam SM, Cornish KM. 2014 The relationship between sleep and behavior in autism spectrum disorder (ASD): a review. J. Neurodev. Disord. **6**, 44. (10.1186/1866-1955-6-44) 25530819 PMC4271434

[3] Patel SP, Cole J, Lau JCY, Fragnito G, Losh M. 2022 Verbal entrainment in autism spectrum disorder and first-degree relatives. Sci. Rep. **12**, 11496. (10.1038/s41598-022-12945-4) 35798758 PMC9262979

[4] Backer van Ommeren T, Vreugdenhil M, Koot HM, Spek A, Scheeren AM, Jertberg RM, Begeer S. 2022 A new real-life test for reciprocity in autistic adults: the interactive drawing test. Front. Psychiatry **13**, 842902. (10.3389/fpsyt.2022.842902) 35386524 PMC8977513

[5] Nayar K, Shic F, Winston M, Losh M. 2022 A constellation of eye-tracking measures reveals social attention differences in ASD and the broad autism phenotype. Mol. Autism **13**, 18. (10.1186/s13229-022-00490-w) 35509089 PMC9069739

[6] Milman NEP, Tinsley CE, Raju RM, Lim MM. 2023 Loss of sleep when it is needed most – consequences of persistent developmental sleep disruption: a scoping review of rodent models. Neurobiol. Sleep Circadian Rhythms **14**, 100085. (10.1016/j.nbscr.2022.100085) 36567958 PMC9768382

[7] Missig G, McDougle CJ, Carlezon WA. 2020 Sleep as a translationally-relevant endpoint in studies of autism spectrum disorder (ASD). Neuropsychopharmacology **45**, 90-103. (10.1038/s41386-019-0409-5) 31060044 PMC6879602

[8] Kondrakiewicz K, Kostecki M, Szadzińska W, Knapska E. 2019 Ecological validity of social interaction tests in rats and mice. Genes Brain Behav. **18**, e12525. (10.1111/gbb.12525) 30311398

[9] Kenkel WM, Gustison ML, Beery AK. 2021 A neuroscientist's guide to the vole. Curr. Protoc. **1**, e175. (10.1002/cpz1.175) 34170636 PMC8244171

[10] McGraw LA, Young LJ. 2010 The prairie vole: an emerging model organism for understanding the social brain. Trends Neurosci. **33**, 103-109. (10.1016/j.tins.2009.11.006) 20005580 PMC2822034

[11] Young KA, Gobrogge KL, Liu Y, Wang Z. 2011 The neurobiology of pair bonding: insights from a socially monogamous rodent. Front. Neuroendocrinol. **32**, 53-69. (10.1016/j.yfrne.2010.07.006) 20688099 PMC3012750

[12] Jones CE, Opel RA, Kaiser ME, Chau AQ, Quintana JR, Nipper MA, Finn DA, Hammock EAD, Lim MM. 2019 Early-life sleep disruption increases parvalbumin in primary somatosensory cortex and impairs social bonding in prairie voles. Sci. Adv. **5**, eaav5188. (10.1126/sciadv.aav5188) 30729165 PMC6353622

[13] DeVries AC, Johnson CL, Carter CS. 1997 Familiarity and gender influence social preferences in prairie voles (*Microtus ochrogaster*). Can. J. Zool. **75**, 295-301. (10.1139/z97-037)

[14] Getz LL, Carter CS, Gavish L. 1981 The mating system of the prairie vole, *Microtus ochrogaster*: field and laboratory evidence for pair-bonding. Behav. Ecol. Sociobiol. **8**, 189-194. (10.1007/BF00299829)

[15] Insel TR, Preston S, Winslow JT. 1995 Mating in the monogamous male: behavioral consequences. Physiol. Behav. **57**, 615-627. (10.1016/0031-9384(94)00362-9) 7777594

[16] Williams JR, Catania KC, Carter CS. 1992 Development of partner preferences in female prairie voles (*Microtus ochrogaster*): the role of social and sexual experience. Horm. Behav. **26**, 339-349. (10.1016/0018-506X(92)90004-F) 1398553

[17] Beery AK, Christensen JD, Lee NS, Blandino KL. 2018 Specificity in sociality: mice and prairie voles exhibit different patterns of peer affiliation. Front. Behav. Neurosci. **12**, 50. (10.3389/fnbeh.2018.00050) 29615879 PMC5868120

[18] DeVries AC, Carter CS. 1999 Sex differences in temporal parameters of partner preference in prairie voles (*Microtus ochrogaster*). Can. J. Zool. **77**, 885-889. (10.1139/z99-054)

[19] DeVries AC, DeVries MB, Taymans SE, Carter CS. 1996 The effects of stress on social preferences are sexually dimorphic in prairie voles. Proc. Natl Acad. Sci. USA **93**, 11 980-11 984. (10.1073/pnas.93.21.11980) PMC38169 8876248

[20] DeVries AC, DeVries MB, Taymans S, Carter CS. 1995 Modulation of pair bonding in female prairie voles (*Microtus ochrogaster*) by corticosterone. Proc. Natl Acad. Sci. USA **92**, 7744-7748. (10.1073/pnas.92.17.7744) 7644488 PMC41222

[21] Anderson DJ, Perona P. 2014 Toward a science of computational ethology. Neuron **84**, 18-31. (10.1016/j.neuron.2014.09.005) 25277452

[22] Pereira TD, Shaevitz JW, Murthy M. 2020 Quantifying behavior to understand the brain. Nat. Neurosci. **23**, 1537-1549. (10.1038/s41593-020-00734-z) 33169033 PMC7780298

[23] Datta SR, Anderson DJ, Branson K, Perona P, Leifer A. 2019 Computational neuroethology: a call to action. Neuron **104**, 11-24. (10.1016/j.neuron.2019.09.038) 31600508 PMC6981239

[24] Mathis A, Mamidanna P, Cury KM, Abe T, Murthy VN, Mathis MW, Bethge M. 2018 DeepLabCut: markerless pose estimation of user-defined body parts with deep learning. Nat. Neurosci. **21**, 1281-1289. (10.1038/s41593-018-0209-y) 30127430

[25] Mathis MW, Mathis A. 2020 Deep learning tools for the measurement of animal behavior in neuroscience. Curr. Opin. Neurobiol. **60**, 1-11. (10.1016/j.conb.2019.10.008) 31791006

[26] Lauer J et al. 2022 Multi-animal pose estimation, identification and tracking with DeepLabCut. Nat. Methods **19**, 496-504. (10.1038/s41592-022-01443-0) 35414125 PMC9007739

[27] Pereira TD et al. 2022 SLEAP: a deep learning system for multi-animal pose tracking. Nat. Methods **19**, 486-495. (10.1038/s41592-022-01426-1) 35379947 PMC9007740

[28] Bohnslav JP et al. 2021 DeepEthogram, a machine learning pipeline for supervised behavior classification from raw pixels. Elife **10**, e63377. (10.7554/eLife.63377) 34473051 PMC8455138

[29] Romero-Ferrero F, Bergomi MG, Hinz RC, Heras FJH, De Polavieja GG. 2019 idtracker.ai: tracking all individuals in small or large collectives of unmarked animals. Nat. Methods **16**, 179-182. (10.1038/s41592-018-0295-5) 30643215

[30] Scribner JL et al. 2020 A neuronal signature for monogamous reunion. Proc. Natl Acad. Sci. USA **117**, 11 076-11 084. (10.1073/pnas.1917287117) PMC7245077 32381740

[31] Lakshmanan A. In press. Automatic annotation of social behavior of voles.

[32] Insel TR, Hulihan TJ. 1995 A gender-specific mechanism for pair bonding: oxytocin and partner preference formation in monogamous voles. Behav. Neurosci. **109**, 782-789. (10.1037/0735-7044.109.4.782) 7576222

[33] Wallace G, Elden M, Boucher R, Phelps S. 2022 An automated radiotelemetry system (ARTS) for monitoring small mammals. Methods Ecol. Evol. **13**, 976-986. (10.1111/2041-210X.13794)

[34] Jones-Tinsley CE et al. 2023 Early life sleep disruption has long lasting, sex specific effects on later development of sleep in prairie voles. Neurobiol. Sleep Circadian Rhythms **14**, 100087. (10.1016/j.nbscr.2022.100087) 36712905 PMC9879777

[35] Wang B et al. 2016 Developmental trajectories of sleep problems from childhood to adolescence both predict and are predicted by emotional and behavioral problems. Front. Psychol. **7**, 1874. (10.3389/fpsyg.2016.01874) 27990129 PMC5131000

[36] Tarokh L, Saletin JM, Carskadon MA. 2016 Sleep in adolescence: physiology, cognition and mental health. Neurosci. Biobehav. Rev. **70**, 182-188. (10.1016/j.neubiorev.2016.08.008) 27531236 PMC5074885

[37] Lord JS, Gay SM, Harper KM, Nikolova VD, Smith KM, Moy SS, Diering GH. 2022 Early life sleep disruption potentiates lasting sex-specific changes in behavior in genetically vulnerable Shank3 heterozygous autism model mice. Mol. Autism **13**, 35. (10.1186/s13229-022-00514-5) 36038911 PMC9425965

[38] Mirmiran M, Scholtens J, Van de Poll NE, Uylings HBM, Van der Gugten J, Boer GJ. 1983 Effects of experimental suppression of active (REM) sleep during early development upon adult brain and behavior in the rat. Brain Res. **283**, 277-286. (10.1016/0165-3806(83)90184-0) 6850353

[39] Feng P, Ma Y. 2003 Instrumental REM sleep deprivation in neonates leads to adult depression-like behaviors in rats. Sleep **26**, 990-996. (10.1093/sleep/26.8.990) 14746380

[40] Bian W-J, Brewer CL, Kauer JA, de Lecea L. 2022 Adolescent sleep shapes social novelty preference in mice. Nat. Neurosci. **25**, 912-923. (10.1038/s41593-022-01076-8) 35618950 PMC9283223

[41] Kayser MS, Yue Z, Sehgal A. 2014 A critical period of sleep for development of courtship circuitry and behavior in *Drosophila*. Science **344**, 269-274. (10.1126/science.1250553) 24744368 PMC4479292

[42] Spieth HT. 1974 Courtship behavior in *Drosophila*. Annu. Rev. Entomol. **19**, 385-405. (10.1146/annurev.en.19.010174.002125) 4205689

[43] Gavish L, Sue Carter C, Getz LL. 1983 Male-female interactions in prairie voles. Anim. Behav. **31**, 511-517. (10.1016/S0003-3472(83)80073-6)

[44] Graham BM, Solomon NG, Noe DA, Keane B. 2016 Male prairie voles with different *avpr1a* microsatellite lengths do not differ in courtship behaviour. Behav. Processes **128**, 53-57. (10.1016/j.beproc.2016.04.006) 27083501

[45] Eban-Rothschild A, Bloch G. 2012 Social influences on circadian rhythms and sleep in insects. In Advances in genetics, pp. 1-32. Cambridge, MA: Elsevier. 10.1016/B978-0-12-387687-4.00001-5 22902124

[46] Kohyama J. 2014 The possible long-term effects of early-life circadian rhythm disturbance on social behavior. Expert Rev. Neurother. **14**, 745-755. (10.1586/14737175.2014.927735) 24902476

[47] Getz LL. 2009 Circadian activity rhythm and potential predation risk of the prairie vole, *Microtus ochrogaster*. Southwest. Nat. **54**, 146-150. (10.1894/PS-40.1)

[48] Gerkema MP, Groos GA, Daan S. 1990 Differential elimination of circadian and ultradian rhythmicity by hypothalamic lesions in the common vole, *Microtus arvalis*. J. Biol. Rhythms **5**, 81-95. (10.1177/074873049000500201) 2133128

[49] Amadei EA et al. 2017 Dynamic corticostriatal activity biases social bonding in monogamous female prairie voles. Nature **546**, 297-301. (10.1038/nature22381) 28562592 PMC5499998

[50] Jones CE et al. 2021 Early life sleep disruption alters glutamate and dendritic spines in prefrontal cortex and impairs cognitive flexibility in prairie voles. Curr. Res. Neurobiol. **2**, 100020. (10.1016/j.crneur.2021.100020) 35505895 PMC9060254

[51] Smeltzer MD, Curtis JT, Aragona BJ, Wang Z. 2006 Dopamine, oxytocin, and vasopressin receptor binding in the medial prefrontal cortex of monogamous and promiscuous voles. Neurosci. Lett. **394**, 146-151. (10.1016/j.neulet.2005.10.019) 16289323

[52] Beery AK, Loo TJ, Zucker I. 2008 Day length and estradiol affect same-sex affiliative behavior in the female meadow vole. Horm. Behav. **54**, 153-159. (10.1016/j.yhbeh.2008.02.007) 18387611 PMC2501115

[53] Lewis R, Curtis JT. 2016 Male prairie voles display cardiovascular dipping associated with an ultradian activity cycle. Physiol. Behav. **156**, 106-116. (10.1016/j.physbeh.2016.01.012) 26780151 PMC4753128

[54] Bueno-Junior LS, Jones-Tinsley CE, Milman NEP, Wickham PT, Watson BO, Lim MM. 2023 Data from: Early-life sleep disruption impairs subtle social behaviours in prairie voles: a pose-estimation study. Dryad Digital Repository. (10.5061/dryad.15dv41p1n) PMC10336370 37448475

[55] Bueno-Junior LS, Jones-Tinsley CE, Milman NEP, Wickham PT, Watson BO, Lim MM. 2023 Early-life sleep disruption impairs subtle social behaviours in prairie voles: a pose-estimation study. Figshare. (10.6084/m9.figshare.c.6728921) PMC10336370 37448475

